# Nuclear warfare: pathogen manipulation of the nuclear pore complex and nuclear functions

**DOI:** 10.1128/mbio.01940-24

**Published:** 2025-03-20

**Authors:** Brianna Steiert, Mary M. Weber

**Affiliations:** 1 Department of Microbiology and Immunology, University of Iowa Carver College of Medicine 12243 , Iowa City, Iowa, USA; 2 Department of Veterinary Microbiology and Pathology, Washington State University 312980 , Pullman, Washington, USA; The Ohio State University, Columbus, Ohio, USA

**Keywords:** NPC, nucleus, bacteria, virus, effector

## Abstract

Viruses and bacteria exploit the nuclear pore complex (NPC) and host nuclear functions to bypass cellular barriers and manipulate essential processes. Viruses frequently engage directly with NPC components, such as nucleoporins, to enable genome import and evade immune defenses. In contrast, bacterial pathogens rely on secreted effector proteins to disrupt nuclear transport and reprogram host transcription. These strategies reflect a remarkable evolutionary convergence, with both types of pathogens targeting the NPC and nuclear functions to promote infection. This minireview explores the overlapping and unique mechanisms by which pathogens hijack the host nucleus, shedding light on their roles in disease and potential avenues for therapeutic intervention.

## INTRODUCTION

Viruses and bacteria exploit the nuclear pore complex (NPC) and nuclear functions to overcome cellular barriers and manipulate host processes. Viral strategies often involve a direct interaction with NPC components, such as nucleoporins, to facilitate genome import and immune evasion (1). Bacterial pathogens, on the other hand, employ secreted effector proteins to disrupt nuclear transport and alter transcriptional landscapes (2, 3). These mechanisms underscore convergent evolutionary pressures to target the NPC and nuclear functions for pathogenic success. This minireview highlights the shared and distinct strategies pathogens use to hijack the host nucleus, offering insights into their roles in infection and potential therapeutic targets.

Compartmentalization is a defining characteristic of eukaryotic cells, allowing for the spatial organization of various biological processes with specialized organelles. This segregation, while beneficial for isolating specific cellular processes, introduces logistical complexities, particularly in moving molecules across membrane barriers to their intended destinations. The movement of materials between the nucleus and cytoplasm, known as nucleocytoplasmic exchange, is vital for transcription and translation (4). The nuclear import of chromatin-binding proteins, such as histones and transcriptional factors, is necessary for transcription, and the nuclear export of mRNA and of ribosomal subunits are requisite steps for ribosome biogenesis and translation (5).

This complexity allows for multiple levels of regulation to maintain homeostasis within the cells. However, this also introduces a myriad of targets for viral and bacterial pathogens. Viruses and bacteria employ diverse yet occasionally overlapping strategies to manipulate host nuclear functions. Viruses, such as herpesviruses and retroviruses, often directly exploit the host’s nuclear transport machinery to facilitate the import of viral genomes and proteins into the nucleus, essential for replication and transcription. For instance, herpesviruses interact with nucleoporins to dock their capsids at nuclear pores (6–9), while retroviruses, like HIV-1, utilize specific host nuclear pore proteins to translocate their core into the nucleus (10–14). On the other hand, intracellular bacteria, including *Chlamydia trachomatis* and *Orientia tsutsugamushi*, secrete effector proteins that disrupt host nuclear transport indirectly, targeting nuclear components such as transcription factors or nuclear lamins to dampen immune responses or alter the gene expression (15–17).

Despite these differences, a striking similarity lies in their exploitation of the host’s nuclear machinery to subvert immune defenses and favor the pathogen’s replication. Both viruses and bacteria target nucleoporins and transport factors like Rae1 and NUP98, with viral proteins such as SARS-CoV-2 ORF6 and bacterial effectors like *Chlamydia*’s CT584 illustrating a conserved strategy to perturb nucleocytoplasmic transport (17–21). These parallels underscore a shared evolutionary pressure to adapt to the logistical and defensive complexities of the host cell nucleus. This review will highlight these overlapping and distinct mechanisms, exploring how viruses and bacteria manipulate nuclear transport, lamina structure, transcription, and chromatin dynamics to achieve their respective pathogenic goals.

## DISRUPTION OF NUCLEOCYTOPLASMIC TRANSPORT BY VIRUSES AND BACTERIA

**Fig 1:**
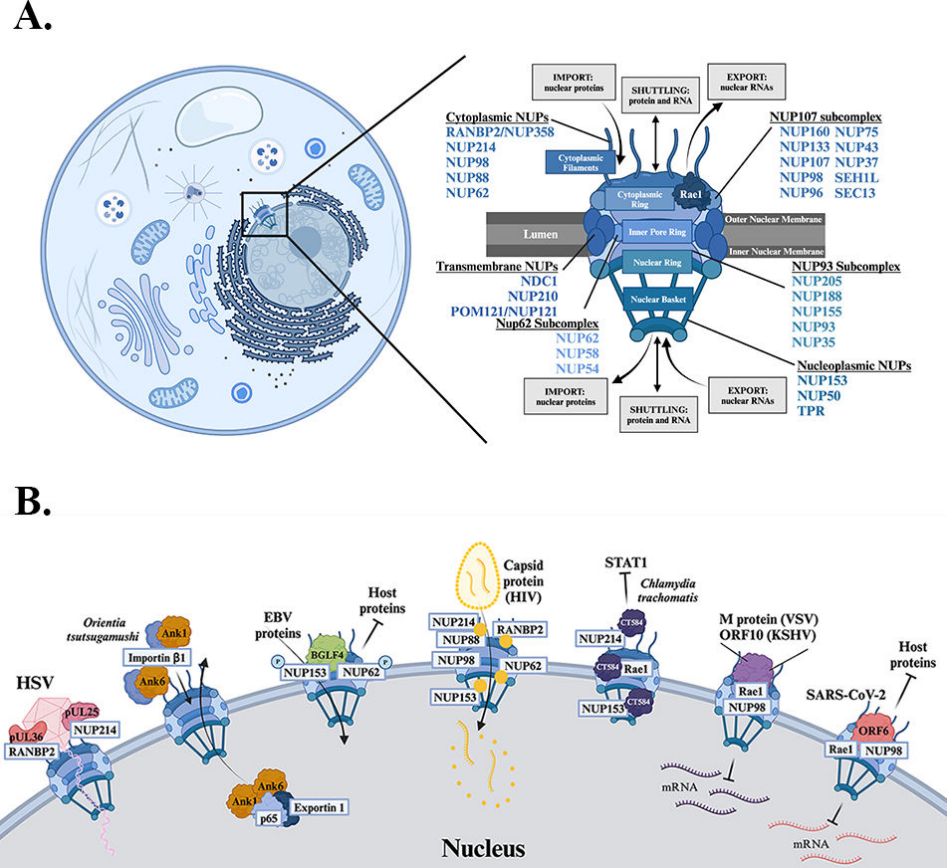
Viruses and bacteria target the nuclear pore complex. (**A**) Schematic depicting the nuclear pore complex, highlighting the core elements, including localization of various NUPs. (**B**) HSV, EBV, HIV, VSV, KSHV, SARS-CoV-2, *Chlamydia trachomatis,* and *Orientia tsutsugamushi* all target the NPC. Left to right: HSV proteins pUL36 and pUL25 target RANBP2 and NUP214, respectively, to allow for uptake of the viral genome into the nucleus. *O. tsutsugamushi* proteins Ank6 and Ank1 are translocated to the nucleus through interactions with importin β1. In the nucleus, these proteins interact with exportin 1 to export p65, preventing NF-κB-mediated transcription. EBV protein BGLF4 phosphorylates NUP153 and NUP62, inhibiting the import of host proteins but allowing for the import of EBV proteins. The HIV capsid protein interacts with multiple NPC proteins, including RANBP2, NUP214, NUP88, NUP62, NUP98, and NUP153. These interactions allow for the translocation of the viral core and subsequent release of viral RNA in the nucleus. *C. trachomatis-*secreted effector CT584 interacts with various NUPs, including NUP214 and NUP153, as well as mRNA export factor Rae1. CT584 is able to impede import of STAT1 into the nucleus following immune stimulation. The M protein of VSV, ORF10 of KSHV, and ORF6 of SARS-CoV-2 all interact with the Rae1–NUP98 complex to block mRNA export, while ORF6 also prevents host protein import.

The nuclear envelope is a double-membrane structure that serves as a critical barrier between the nucleus and the cytoplasm, regulating the flow of molecules between these two compartments. Nuclear pore complexes (NPCs) are intricate structures that bridge the inner and outer nuclear membranes, creating a 50-nm-wide channel for controlled exchange of materials between the cytoplasm and nucleus (4, 22). Each NPC is a massive macromolecular assembly (~120 MDa in humans) composed of 8–64 copies of 30 distinct nuclear pore proteins, known as nucleoporins (NUPs), that fuse the inner and outer nuclear membranes to form a channel for shuttling molecules between the nucleus and cytoplasm (23) (Fig. 1A). NUPs can be broadly divided into scaffold NUPs, which primarily serve structural roles, and FG NUPs, which contain intrinsically disordered domains rich in phenylalanine–glycine (FG) repeats (FG, FXFG, or GLFG) (24, 25). These FG NUPs line the channel, forming a disordered matrix of proteins that effectively acts as a sieve to control passive transport (26, 27). FG NUPs also tightly control active transport through the NPC, guiding proteins and mRNP complexes as they pass from one FG repeat to the next within the channel. With each FG NUP containing 5–50 repeat regions and with nearly 200 FG NUPs in the NPC, this creates over 1,000 potential binding sites for transport factors. This complex architecture allows for the exchange of diverse molecules, from large complexes such as viral capsids, which are transported intact and fully folded, to small molecules such as nucleotides. While small proteins (<40 kDa) can passively diffuse through the NPC, larger proteins and complexes require active transport.

Nuclear transport factors (NTFs), also known as karyopherins, facilitate nucleocytoplasmic transport by mediating interactions between cargo and FG NUPs (4, 28–30). Karyopherins bind to specific signal sequences in cargo proteins, with importins recognizing nuclear localization signals (NLS) in the cytoplasm and exportins binding to nuclear export signals (NES) in the nucleoplasm. The assembly and disassembly of these complexes is tightly regulated by the small GTPase Ran. In the nucleus, RanGTP promotes cargo release from importins. During nuclear export, RanGTP binds to exportins, driving complex formation with cargo, and is hydrolyzed in the cytoplasm, leading to dissociation of the export complex. RanGDP is then recycled to the nucleoplasm by nuclear transport factor 2 (NTF2), where the RanGEF regulator of chromosome condensation 1 (RCC1) catalyzes the exchange of GDP for GTP, regenerating the pool of RanGTP. While there are several isoforms and types of NTFs, each recognizing different cargo, significant redundancy exists among them. The mechanisms by which cargo-NTF specificity is conferred remain unknown, but it may involve recognition of specific domain folds, localization signals, or occur via indirect interactions with other factors (28, 31–34).

As essential components of the NPC, NUPs serve as gatekeepers that regulate the bidirectional transport of proteins, RNAs, and other macromolecules between the nucleus and the cytoplasm. This tightly controlled exchange is crucial for maintaining cellular homeostasis and responding to various signals. Due to their central role in nucleocytoplasmic transport, NUPs and the broader NPC are prime targets for viral manipulation, with many viruses evolving sophisticated mechanisms to hijack the NPC for their own benefit. Some viruses directly interact with specific NUPs to disrupt their normal function, while others target the karyopherin family of transport proteins, altering cargo transport across the nuclear envelope. These strategies enable viruses to exploit the NPC in two key ways. First, by altering nucleocytoplasmic transport, viruses can prevent the proper trafficking of host immune signaling proteins, thus dampening the host’s antiviral response. Second, viruses can use the NPC to gain access to the nucleus, where they can replicate, transcribe their genomes, or integrate their genetic material into the host DNA. By co-opting the cell’s transport machinery, viruses not only evade immune surveillance but also create a favorable environment for their replication. In this section, we focus on the unique and intricate ways in which viruses and bacteria hijack NUPs and the NPC and offer insights into how this promotes replication and spread.

Herpes simplex virus (HSV) is a double-stranded DNA virus that directly interacts with the NPC to facilitate the release of its viral genome into the host cell nucleus. Following fusion of the virion envelope with the host cell membrane, the nucleocapsid (double-stranded viral DNA plus its protective protein shell) and tegument (layer of proteins between the nucleocapsid and envelope unique to herpesviruses) proteins are released into the cytoplasm where they ultimately localize to the cytoplasmic face of the NPC (6). This process is mediated by capsid-tethered tegument proteins pUL36 and pUL25, which bind to RANBP2 (NUP358) and NUP214, respectively (7, 8) (Fig. 1B). It is hypothesized that during this stage, the capsid portal is oriented to the NPC, triggering a conformation change that facilitates the release of the viral genome into the nucleoplasm, where viral replication and transcription are initiated. As newly synthesized nucleocapsids undergo nuclear egress, they are prevented from reattaching to the NPC by the actions of the viral proteins pUL16 and pUL21, which likely form a complex post-infection (9). This mechanism ensures that newly synthesized virions do not re-enter the viral assembly pathway, maintaining efficient viral production and propagation.

The gamma-herpesvirus Epstein–Barr virus (EBV) uses a Ser/Thr protein kinase, BGLF4, which directly binds to NUP62 and NUP153 to reorganize the NPC (35, 36) (Fig. 1B). BGLF4 interacts with FG-rich regions of NUP62 and NUP153, mimicking how the canonical binding partner importin-β interacts with these NUPs to carry the cargo across the channel. Consequently, BGLF4-mediated redistribution of NUP62 and NUP153 impairs nuclear import of host NLS-containing proteins while simultaneously promoting the import of non-NLS-containing EBV proteins (36). Phosphorylation of these FG NUPs by BGLF4 leads to pore dilation, which may serve as a mechanism to block the import of host factors while promoting the import of viral proteins.

Retroviruses, such as human immunodeficiency virus (HIV), must not only enter the nucleus of the host cell but also must integrate their genomic information into the host chromatin. To accomplish this, the viral core must cross the nuclear envelope, which is enabled by NUP153 and NUP358 (RANBP2). The capsid protein (CA) has been shown to interact with multiple nucleoporins, including those associated with the cytoplasmic filament (NUP358, NUP214, and NUP88), inner ring (NUP62 and NUP98), and nuclear basket (NUP153) (10) (Fig. 1B). Depletion of NUP358 or NUP153 perturbed nuclear import (11–13), leading to a model in which NUP358-CA binding enables capsid docking at the NPC (11, 12), while high-affinity interactions between CA hexamers and NUP153 orient it for passage through the NPC (14). Infection induces a marked change in NUP abundance, notably of NUP62 (37), which may reduce the barrier properties associated with the NPC to promote import (38).

## TARGETING THE NPC TO DAMPEN THE HOST IMMUNE RESPONSE

Upon viral infection, the host mounts an innate immune response, which involves the activation and nuclear translocation of various transcription factors. Transcription factors such as interferon-regulatory factor 3 (IRF3)**,** IRF7**,** and nuclear factor kappa-light-chain-enhancer of activated B cells (NF-κB) are transported into the nucleus to initiate transcription of innate immune response genes, including type I interferons (IFN) and proinflammatory cytokines (39). The immune response is further amplified by the translocation of transcription factors such as IRF9 and STAT1/2, which induce the expression of interferon-stimulated genes (ISGs) that work to suppress viral replication. Importantly, the nuclear translocation of IRF3, NF-κB, and STAT1/2 is dependent on specific importins and NPC components (40). Specifically, as discussed below, targeting of the Rae1–NUP98 complex has evolved as a common method used by multiple viruses to downregulate the host’s antiviral response through blocking transcription factor import and/or inhibiting export of mRNA transcripts (18–20, 41).

Besides being one of the key structural components associated with the viral capsid, the vesicular stomatitis virus matrix protein (VSV M protein) binds to both NUP98 and Rae1 (42, 43), thereby impeding mRNA export, transcription, and translation (Fig. 1B). While NUP98 is an FG NUP and thus serves as an important ligand for NTFs, it also has a binding pocket for Rae1 (44). Rae1 contains seven WD40 repeats, forming a seven-bladed β-propeller that serves as a platform for protein–protein interactions and RNA binding (44). Crystallization of the M–Rae1–NUP98 complex revealed that M protein binds to the Rae1–NUP98 heterodimer through two projections that extend from its globular domain (45). While both projections connect with the β-propeller of Rae1, contact was also made with NUP98, suggesting a directional interaction with both Rae1 and NUP98. A highly conserved methionine (Met51) fastens into the hydrophobic pocket of Rae1, with acidic residues upstream and downstream of Met51 interacting with basic grooves on Rae1’s surface. Notably, M protein competes with various oligonucleotides for binding to Rae1–NUP98, suggesting that the VSV M protein mimics the phosphate backbone of nucleic acids to bind within this site (45, 46). As mRNAs normally use this site to pass through the nuclear pore tethered to Rae1, M protein binding effectively blocks mRNA export. This not only suppresses the host’s antiviral response but also enables the virus to hijack the host’s protein synthesis machinery for the production of viral proteins.

Since this initial discovery in VSV, other viruses have been found to target the Rae1–NUP98 complex, suggesting that interference with this complex is a conserved viral strategy to subvert host defenses. ORF10 of Kaposi’s sarcoma-associated herpesvirus induces an accumulation of poly(A)+ mRNA in the nucleus, indicating reduced mRNA export (41). Global mapping of the herpesvirus–host interactome identified Rae1 and NUP98 as potential interacting partners of ORF10 (47) (Fig. 1B). Co-immunoprecipitation confirmed that ORF10 readily binds to Rae1; however, the interaction with NUP98 was minimal in the absence of Rae1, suggesting that ORF10 likely interacts with NUP98 through Rae1 (41). While the ORF10–Rae1–NUP98 complex shares structural similarities with that formed by the VSV M protein, a key distinction lies in the fact that RNA is still able to interact with the complex via ORF10 (48). This unique interaction allows ORF10 to selectively block cellular mRNAs based on their 3’ untranslated region (UTR), rather than causing a global export block, as is the case with VSV M protein. This nuanced interaction highlights a unique regulatory mechanism that could be used to promote viral propagation while facilitating evasion of immune detection. Further research is needed to dissect how this selectivity is achieved and whether other host or viral factors may contribute to this process.

Recently, ORF6 from SARS-CoV-2 was also shown to directly interact with the Rae1–NUP98 complex, leading to a block in nuclear import of a broad range of host proteins and trapping mRNAs, especially those derived from ISGs, in the nucleus (18–21) (Fig. 1B). Direct interactions between ORF6 and Rae1–NUP98 are proposed to obstruct the NPC channel, impairing the docking of cargo—karyopherin family receptor protein complexes. Notably, binding of ORF6 to Rae1–NUP98, and its ability to impede nucleocytoplasmic transport, similarly requires a C-terminal conserved methionine (Met58). This methionine extends into the Rae1 mRNA-binding groove while flanking acidic residues form salt-bridges with Rae1 (18, 20, 49). Additionally, variants of ORF6 (BA.2 and BA.4) that harbor a D61L point mutation are impaired in interacting with Rae1–NUP98. This mutation results in elevated nuclear translocation of STAT2, a diminished ability to block mRNA export, and attenuated pathogenesis in Syrian hamsters (19). Collectively, this supports a model in which reducing innate immune antagonism is linked to ORF6 function, highlighting that Rae1 and NUP98 serve as conserved targets for viruses across various families, encompassing both RNA and DNA viruses.

While initially associated with viral interactions, multiple NUPs and Rae1 were recently shown to be the target of CT584 from the obligate intracellular bacterium *Chlamydia trachomatis* (17). Although the interacting NUPs are dispersed throughout the NPC, they are all classified as FG NUPs, suggesting that CT584 may interact with them through a mechanism similar to that of canonical binding partners or cargo transported through the NPC. Interestingly, ectopically expressed CT584 can reduce the nuclear import of STAT1 following interferon-gamma (IFNγ) induction (Fig. 1B). While the precise mechanism of this inhibition remains unclear, it is possible that CT584 may perturb nucleocytoplasmic transport in a mechanism similar to ORF6 from SARS-CoV-2 or VSV M protein. Moreover, CT584’s localization to the nuclear envelope of adjacent bystander cells raises intriguing questions about its potential role in intercellular communication during infection. The ability of CT584 to translocate into neighboring cells may allow *C. trachomatis* to manipulate host cell processes beyond the initially infected cell, priming these adjacent cells for subsequent infection. Understanding these parallels between bacterial and viral manipulation of nucleocytoplasmic transport could provide valuable insights into the broader mechanisms of host–pathogen interactions.

*Orientia tsutsugamushi*, the causative agent of scrub typhus, secretes an extensive arsenal of Ankyrin (Ank) proteins through a type I secretion system, with several shown to localize to the host cell nucleus when ectopically expressed (15). Both Ank1 and Ank6 translocate to the nucleus via importin β1, where they directly bind to the transcription factor p65 and promote its export through interactions with exportin 1 (16) (Fig. 1B). While these proteins do not bind to nuclear pore components like the aforementioned pathogens, they appear to disrupt nucleocytoplasmic transport mechanisms. By reducing nuclear p65 accumulation, *O. tsutsugamushi* effectively dampens the NF-κB signaling pathway, a crucial regulator of antimicrobial gene expression, thereby aiding bacterial infection. Notably, *Orientia* can still inhibit NF-κB-mediated gene transcription even when exportin 1 is inhibited, suggesting that it employs multiple mechanisms to interfere with NF-κB signaling. Interestingly, most *Orientia* Ank proteins, including Ank1 and Ank6, possess C-terminal F-box domains and PRANC domains. The F-box domain binds SKP1 to form the SCF1 complex, indicating a potential involvement of ubiquitination (50, 51). This dual functionality suggests that Ank1 and Ank6 may inhibit NF-κB through a two-pronged approach: by promoting p65 export and potentially targeting p65 or associated proteins for ubiquitination and degradation. Future studies are needed to clarify the precise mechanism(s) by which Ank1 and Ank6 modulate NF-κB signaling. These findings illustrate yet another strategy used by microbial pathogens to subvert host cellular processes.

Collectively, these organisms highlight the evolutionary pressure for pathogens to subvert the NPC. The nucleus is essential for DNA synthesis, making it a prime target for viruses seeking to replicate their genomes. Further, both viruses and bacteria manipulate translocation across the nuclear pore in both directions—some allowing for translocation of pathogen proteins while blocking host proteins, whereas others block mRNA export. This ultimately dampens the host immune response, particularly the type I interferon and NF-κB pathways.

## MODULATION OF THE NUCLEAR LAMINA BY VIRUSES AND BACTERIA

The nuclear lamina is a thin, dense filamentous network that lies beneath the inner nuclear membrane (INM), providing essential structural support to maintain nuclear shape and spatial organization. Beyond its architectural role, the lamina serves as a dynamic regulator of key nuclear processes, including the organizing of nuclear pore complexes, regulation of gene expression, DNA repair, and mRNA splicing (52–54). Structurally, the lamina is composed of type V intermediate filament proteins, including A-type lamins (lamins A and C) and B-type lamins (lamin B1 and B2), which together form a robust cross-linked network. These lamins are anchored to the INM through interactions with lamin-associated proteins (LAPs) such as emerin and the lamin B receptor, ensuring the stability of this vital scaffold and connecting it to chromatin (55). Given its roles in both nuclear integrity and cellular function, the lamina is a frequent target for pathogens seeking to subvert host cellular processes.

**Fig 2:**
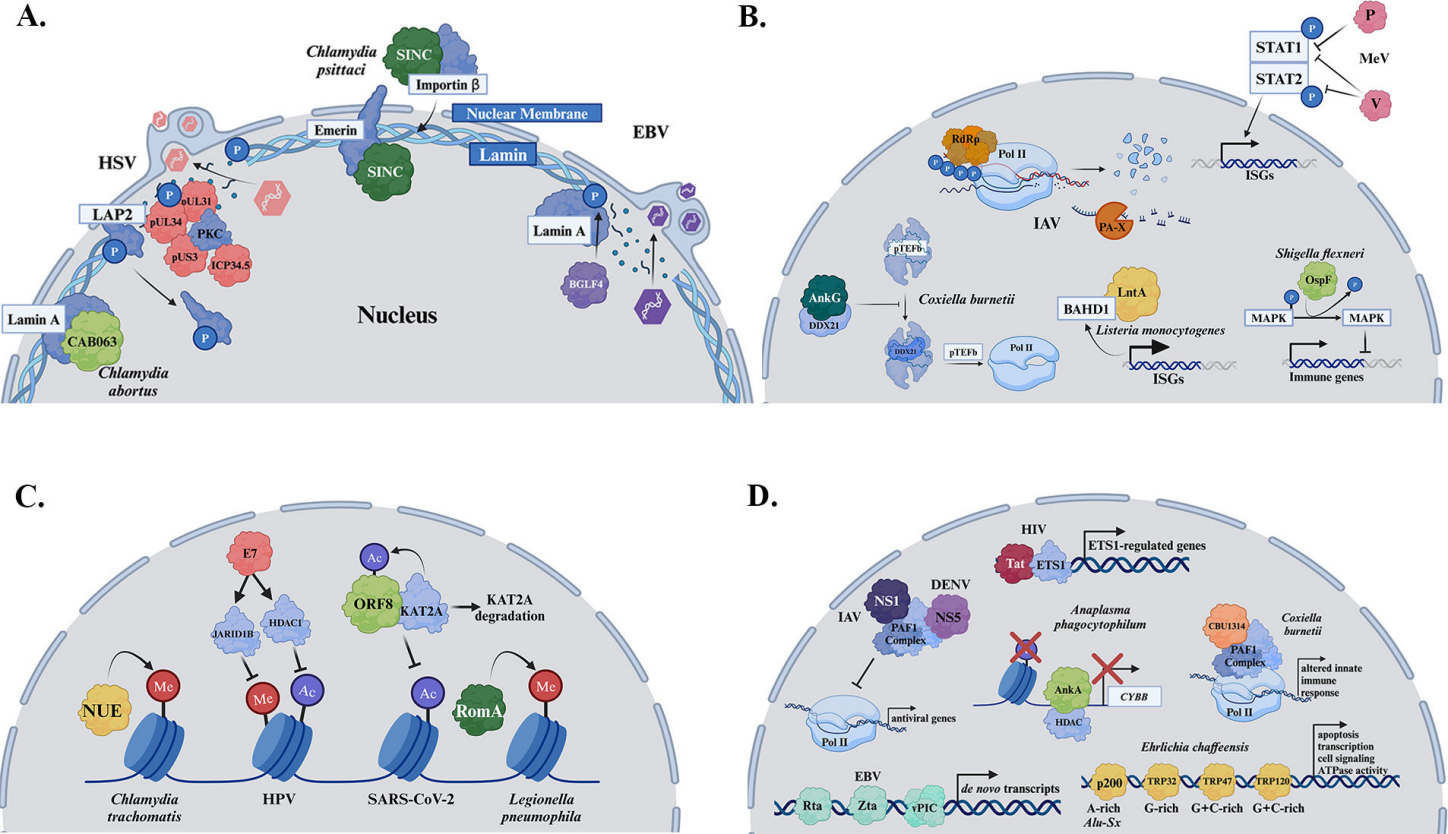
Pathogens target the nuclear lamin, transcriptional regulators, histones, and DNA. (**A**) EBV, HSV, *Chlamydia abortus,* and *Chlamydia psittaci* all target the nuclear lamin. Left to right: *C. abortus* protein CAB063 binds to lamin A. The consequence of this interaction is unknown. HSV-1 infection leads to a redistribution of laminal proteins. Viral proteins pUL31 and pUL34 alter the localization of LAP2. Further, host PKC is recruited, and along with viral proteins pUS3 and ICP34.5, lamin and LAP proteins are phosphorylated, resulting in disruption of the lamin layer. This allows for nuclear egress. *C. psittaci* protein SINC is translocated into the nucleus through interactions with importin β. Inside the nucleus, SINC interacts with the laminal protein emerin. The physiological relevance of this interaction is yet to be determined. The EBV protein BGLF4 interacts with lamin A, resulting in phosphorylation of lamin A and redistribution of the nuclear lamina to help facilitate nuclear egress. (**B**) IAV, MeV, *Coxiella burnetii, Legionella pneumophila,* and *Shigella flexneri* target transcriptional regulators. Left to right: *C. burnetii* protein AnkG interacts with DDX21. This interaction inhibits the canonical function of DDX21, which would normally release pTEFb from the 7SK snRNP complex to initiate transcription with Pol II. This allows *C. burnetii* to control the expression of genes involved in apoptosis, intracellular trafficking, and transcription. IAV interacts with Pol II to alter host transcription. IAV RNA-dependent RNA polymerase (RdRp) interacts with hyperphosphorylated Pol II, causing its degradation. IAV protein PA-X also targets Pol II-specific mRNA transcripts for degradation. *L. monocytogenes* protein LntA binds BAHD1. This displaces BAHD1 from ISG promoters, resulting in upregulation of ISGs. MeV dampens the host immune response by targeting host transcription factors. One example, depicted here, demonstrates how MeV proteins V and P can target the JAK-STAT pathway by preventing the phosphorylation of STAT1 and STAT2, thus inhibiting their translocation into the nucleus and transcription factor activity. *S. flexneri* protein OspF dephosphorylates MAPKs inhibiting MAPK-dependent phosphorylation of histone H3, ultimately altering the expression of immune response genes. (**C**) SARS-CoV-2, HPV*, Legionella pneumophila,* and *Chlamydia trachomatis* target histones. Left to right: *C.t*. protein NUE is a eukaryotic-like SET-domain containing protein with DNA methyltransferase activity. However, the effect on the host transcription landscape is yet to be determined. Viral protein E7 of HPV recruits HDAC1 and JARID1B to the TLR9 promoter region, inhibiting their ability to acetylate and methylate histones. ORF8 of SARS-CoV-2 is a histone mimic, usurping host enzyme KAT2A away from its canonical targets. This results in acetylation of ORF8, degradation of KAT2A, and reduced H3 acetylation. *L. pneumophila-*secreted effector RomA also contains a eukaryotic-like SET domain with methyltransferase activity. It can methylate histone 3 lysine 14, repressing gene expression. (**D**) EBV, HIV, IAV, DENV, *Coxiella burnetii*, *Anaplasma phagocytophilum,* and *Ehrlichia chaffeensis* target DNA directly. Left to right: IAV protein NS1 and DENV protein NS5 both bind the PAF1 complex to prevent its interaction with Pol II, inhibiting the transcription of antiviral genes. EBV transcription factors, Zta and Rta, and viral preinitiation complex (vPIC) bind unique sites on the host genome, resulting in “*de novo”* transcripts that alter the host transcriptional landscape. *A. phagocytophilum* effector protein AnkA binds AT-rich host DNA where it acts to downregulate the expression of host gene *CYBB* with the help of recruitment of HDAC1 to deacetylate histone H3. One of the functions of HIV protein Tat is to directly interact with host DNA. Tat interacts with transcription factor ETS1 at promoter sites to alter the expression of ETS1-regulated genes. *E. chaffeensis* secretes multiple proteins that bind various regions of host DNA including p200 (A-rich Alu-Sx elements), TRP32 (G-rich), TRP47 (G + C rich), and TRP120 (G + C rich), leading to altered transcription. *C. burnetii* effector CBU1314 binds to the host PAF1 complex, which is important for Pol II regulation. This interaction leads to an altered innate immune response.

HSV-1 infection induces alterations in nuclear shape and lamin protein redistribution, facilitating capsid docking at the INM and ensuring there is an adequate area of flexible membrane available to encapsulate the capsid. To accomplish this, HSV-1 targets and redistributes local lamin subunits, disrupting nuclear organization to support viral egress (56, 57) (Fig. 2A). Previous studies show that co-expression of viral proteins pUL31 and pUL34 causes the mislocalization of lamin-associated protein 2 (LAP2), leading to the redistribution and reduction of lamin A/C (58). More recent findings suggest that manipulation of the INM relies on the nuclear egress complex (NEC), which recruits both cellular protein kinase C (PKC) and viral protein kinase pUS3. In part through interactions with the viral protein ICP34.5, these kinases phosphorylate lamins and LAPs, promoting nuclear lamina disassembly and viral de-envelopment (59–61). Additional HSV-1 proteins, ICP22 and pUL47, have been shown to interact with emerin and lamin A/C, though the exact impact of these interactions remains unknown (62, 63). These coordinated disruptions of the nuclear lamina underscore the importance of this structure in host defense and highlight its role as a strategic target for viral manipulation.

Epstein–Barr virus (EBV), a member of the herpesvirus family, interacts with nuclear lamins to promote virion production and control latency. BGLF4, previously identified as a target of nucleoporins, also interacts with lamins A/C (Fig. 2A). Phosphorylation of lamin A induces disassembly and redistribution of the nuclear lamina, as observed by microscopy (64). Mutation of the five phosphorylated serine residues in lamin A to alanine residues significantly reduces virion production in EBV-infected cells, suggesting that EBV-induced phosphorylation of lamin A facilitates nuclear egress (64). Interestingly, lamins A/C may also play a role in restricting EBV gene expression during latency. In latently infected B cells, lamin A/C expression is upregulated in cells adopting Type III latency compared to Type I latency (65). Lamins are known to bind genomic DNA and alter chromatin structure, thereby influencing gene expression. Recent studies have demonstrated a similar role in latent EBV infection, such that the interaction between EBV DNA and nuclear lamins results in increased expression of lytic replication genes (65). Knockout of lamin A/C gene *LMNA* resulted in decreased expression of lytic replication genes and increased expression of Type III latency genes (65). These findings collectively underscore the critical role of the nuclear lamina in regulating lytic replication and maintaining the balance between latency and reactivation in EBV infection.

*Chlamydia psittaci*, a zoonotic pathogen that can be spread from avian species to humans, encodes a type-III secreted effector protein known as secreted inner nuclear membrane-associated *Chlamydia* protein (SINC), which localizes to the nuclear envelope (66, 67) (Fig. 2A). This localization depends on the NPC and likely involves importin-β, as inhibition of importin-β significantly reduced SINC’s presence at the nuclear envelope. Notably, SINC was shown to localize to the nuclear envelope in both infected and neighboring cells (66). Using a proximity-based *in vivo* biotinylation method, BioID, 22 putative targets of SINC were identified, including laminB1, emerin, LAP1, MAN1, and LBR, of which binding to emerin was confirmed by IP (66). While each of these proteins are critical components of the INM, they also play significant roles in cell signaling, gene regulation, and heterochromatin regulation (68). Additionally, *Chlamydia abortus*, a species that infects ruminants, encodes an SINC ortholog, CAB063, that similarly localizes to the nuclear membrane where it interacts with lamin A (69) (Fig. 2A). Intriguingly, CAB063 induced the formation of lobulated nuclei and increases nucleoli numbers, leading to heightened rates of apoptosis in CAB063 transfected cells (69). Despite these findings, the physiological consequence of these interactions and the specific function of SINC and CAB063 remain undefined, prompting intriguing speculation about their roles.

Serving as a key regulator of NPC organization, gene expression, DNA repair, and mRNA splicing, lamin proteins are common targets for both bacterial and viral pathogens. While viruses primarily manipulate the nuclear lamina to facilitate their transport across the nuclear membrane, EBV also targets lamins to alter the gene expression during latency. Although *Chlamydia* species are known to interact with lamin proteins, the functional consequences of these interactions remain unclear. However, similar to EBV, it is plausible that *Chlamydia* may target lamins to modulate host gene expression.

## PATHOGEN MODULATION OF HOST GENE EXPRESSION THROUGH TRANSCRIPTIONAL REGULATORS

In addition to subverting host transcription machinery for their own genome amplification, viruses and bacteria actively manipulate host gene expression. In the next three sections of this review, we will explore the various mechanisms by which viruses and bacteria alter host gene expression, through interactions with transcriptional regulators, posttranslational modifications, and direct binding of DNA. There is clear evolutionary pressure for pathogens to suppress host immune responses and evade clearance. These host–pathogen interactions ultimately lead to the downregulation or suppression of antimicrobial response, converging on key pathways related to interferon signaling, inflammation, apoptosis, and others that remain to be discovered.

Influenza A virus (IAV) targets host RNA polymerase II (Pol II) as a strategy to evade the host antiviral defense system (70) (Fig. 2B). IAV’s RNA-dependent RNA polymerase interacts with the large subunit of hyperphosphorylated Pol II (71), leading to its subsequent degradation. Notably, this degradation coincides with reduced host transcription and the onset of viral replication (72). Furthermore, Pol II-specific mRNA transcripts are targeted by IAV protein PA-X for degradation, further suppressing the antiviral response to infection (73). This illustrates a key mechanism by which a virus can manipulate host gene expression through targeting RNA polymerase II and its transcripts.

Measles virus (MeV) infection elicits an innate immune response; however, the virus has evolved numerous strategies to dampen this response by targeting host transcription factors. Multiple measles proteins (M, P, V, and C) have been implicated in combating the innate immune response and altering host gene expression (74, 75). One notable example is the targeting of the JAK-STAT pathway to inhibit the expression of ISGs. Following infection, IFN-β is produced and binds to type-I IFN receptors, leading to the activation of the JAK-STAT pathway. In this pathway, JAK family kinases phosphorylate STAT family proteins, which then complex with IRF9 to translocate to the nucleus, where they act as transcription factors to upregulate ISG expression (75). During infection, MeV proteins can interrupt this pathway at multiple points to reduce ISG expression (Fig. 2B). For instance, the V protein binds STAT2, inhibiting its function (76, 77). Additionally, the P and V proteins prevent the phosphorylation of STAT1, thereby blocking its nuclear translocation and transcription factor activity (78, 79). These redundant mechanisms result in blocking transcription of ISGs. This highlights another strategy by which viruses target host transcription, specifically through direct interference of host transcription factors.

Much like viruses, bacteria have evolved sophisticated strategies to manipulate host gene expression to their advantage. In the mid-1970s, Van Montagu et al. (80) discovered that *Agrobacterium tumefaciens* releases its tumor-inducing (Ti) plasmid, which includes a segment called T-DNA that integrates into the host genome, inducing uncontrolled cell proliferation and producing nutrients such as opines to support bacterial growth. Since this remarkable example of microbial exploitation of the host nucleus, numerous studies have revealed that various bacterial pathogens target the nucleus to selectively control host gene expression, thereby thwarting the host response to infection. These so-called nucleomodulins represent a growing class of bacterial virulence factors that co-opt chromatin dynamics, histone modifications, DNA methylation, DNA replication, and RNA splicing (2). Here, we highlight a few bacterial effectors that directly engage with host nuclear proteins, with a particular emphasis on those produced by intracellular bacteria. For a detailed review on this topic, see Hanford et al. (3).

During *Listeria monocytogenes* infection, the secreted effector protein *Listeria* nuclear targeted protein A (LntA) enters the nucleus and directly interacts with the chromatin-repressing protein bromo adjacent homology domain-containing 1 (BAHD1) (Fig. 2B). BAHD1 functions as a scaffolding protein that complexes with other chromatin factors to promote chromatin compaction and facilitate heterochromatin formation. By binding to BAHD1, LntA alleviates its association with ISG promoters, leading to chromatin unwinding and upregulation of the interferon response in a type III interferon-dependent manner (81, 82).

*Shigella flexneri* secretes multiple putative nucleomodulins; however, OspF is of particular interest as it uses an atypical NLS that requires importin-α1 to enter the nucleus (83). OspF is a phosphothreonine lysase that modifies host mitogen-activation protein kinase (MAPKs) through removal of phosphate groups from the phosphothreonine residue in the activation loop (84) (Fig. 2B). This unique modification inactivates and locks MAPKs in the nucleus, consequently inhibiting phosphorylation of histone H3 and preventing modification to chromatin structures such that NF-κB promoter regions are no longer exposed, preventing proinflammatory chemokine and cytokine production (85). Additionally, OspF interacts with chromatin reader heterochromatin protein 1 (HP1), interfering with its phosphorylation (86), and allowing the pathogen to fine-tune the host inflammatory response to *Shigella* in the colon.

Large-scale screens, including ectopic expression, have identified bacterial effector proteins that localize to the nucleus (15, 87–91). The zoonotic pathogen *Coxiella burnetii* secretes a substantial repertoire of T4SS effector proteins into the host cell during infection, and a subset of these utilize classical or nonclassical NLS to target the nucleus. To date, eight effectors have been shown to localize to the nucleus; however, only AnkG, CBU1314, NopA, and Cae1 have characterized functions (see below for CBU1314 and NopA). Similar to many other intracellular pathogens, *C. burnetii* modulates apoptosis in host cells to enhance its survival and prolong infection. AnkG was initially shown to block pathogen-induced apoptosis through interactions with p32 (92). This effect depends on AnkG’s nuclear localization and its interactions with both RNA helicase DDX21 and the 7SK small nuclear ribonucleoprotein (7SK snRNP) (93) (Fig. 2B). By preventing DDX21 translocation from the nucleolus to the nucleoplasm, AnkG blocks the release of pTEFb from the 7SK snRNP complex, enabling *C. burnetii* to control genes involved in apoptosis, intracellular trafficking, and transcription (93). This emphasis on apoptosis inhibition is further underscored by the nuclear effector CaeA, which also modulates apoptosis, though its mechanism remains to be defined (94). Further investigation into these nuclear effectors will provide valuable insights into the diverse strategies *C. burnetii* employs to manipulate host cellular pathways and promote intracellular infection.

## MODULATION OF HOST GENE EXPRESSION THROUGH POST-TRANSLATIONAL MODIFICATIONS

Gene expression is regulated not only through the binding of transcription factors but also through post-translation modifications of chromatin (95). DNA is compacted into a nucleoprotein complex, called chromatin, whose fundamental unit is the nucleosome. Each nucleosome consists of a histone octamer (two copies each of H2A, H2B, H3, and H4) around which DNA is wound (96). Histones possess various sites for modifications, including acetylation, methylation, phosphorylation, ubiquitination, and sumoylation, collectively known as epigenetic regulation. Specific combinations of these post-translational modifications (PTMs) on the globular core or terminal tails of histones can drive transcriptional activation by inducing local changes in the chromatin structure (95). These epigenetic modifications play a crucial role in regulating gene expression by altering DNA accessibility and recruiting specific protein complexes. Acetylation of histone tails, for instance, generally promotes a more open chromatin structure, facilitating transcription. Conversely, certain methylation patterns can lead to chromatin compaction and gene silencing (97). The intricate interplay between these modifications creates a “histone code” that fine-tunes gene expression in response to various cellular signals and environmental cues. In this section, we highlight a few of the ways bacteria and viruses co-opt these processes to manipulate host gene expression, evade immune responses, and promote their own replication and survival within host cells.

SARS-CoV-2 is a virus that modulates host chromatin to alter gene expression. Infection with SARS-CoV-2 is associated with the suppression of interferon response genes and the upregulation of pro-inflammatory genes, changes that can be mapped to altered chromatin structure (98). Some of these changes can be attributed to a histone mimic encoded by ORF8 of SARS-CoV-2 (Fig. 2C). ORF8 shares homology with the tail region of human histone H3, specifically containing an ARKS motif (99). In humans, ARKS motifs are common targets for acetylation and methylation, modifications that activate or repress gene expression, respectively. During SARS-CoV-2 infection, ORF8 is acetylated by the enzyme KAT2A (99). Interestingly, KAT2A expression decreases after infection, suggesting that ORF8 may promote KAT2A degradation to reduce H3 acetylation. This reduction in acetylation is accompanied by increased chromatin compaction and a consequent decrease in gene expression (99). Notably, strains deficient in *orf8* exhibit reduced viral replication (99), underscoring the importance of histone modification in viral defense mechanisms.

Viruses can also recruit proteins to alter acetylation and methylation, thereby controlling gene expression. During human papillomavirus (HPV) Type 16 infection, histone deacetylase HDAC1 and histone demethylase JARID1B are recruited to the toll-like receptor 9 (TLR9) promoter region, modifying histones upstream of the TLR9 transcriptional start site (100) (Fig. 2C). This recruitment ultimately reduces TLR9 expression and dampens the interferon response to infection. This effect is dependent on the viral protein E7 as silencing this gene restores histone acetylation and methylation (100). These are just a couple of examples of how viruses regulate host genes by altering histone PTM. For a detailed review on this topic, see Li et al. (101).

The SET domain is a 130-residue domain found in histone methyltransferases, which transfer a methyl group to lysine residues within histones using S-adenosyl-L-methionine as the methyl donor (102). Unlike eukaryotes, prokaryotes lack histones; however, some pathogenic bacteria encode proteins with eukaryotic-like domains that could allow them to modulate host gene expression. Sequence analysis of *C. trachomatis* revealed an SET domain in CT737 (103). CT737 was shown to be secreted in a T3SS-dependent manner using *S. flexneri* as a surrogate host and localized to the nucleus when ectopically expressed, earning its designation as a nuclear effector (NUE). Significantly, NUE was shown to localize to the nuclear fraction during infection and was capable of modifying histones H2B, H3, and H4 in *in vitro* enzymatic assays (Fig. 2C)*.* While *Chlamydiae* encode histone-like proteins, Hc1 and Hc2, which play a role in DNA compaction during RB to EB conversion (104–107)*, C. trachomatis* NUE was unable to modify Hc1 (103). Intriguingly, the *C. pneumoniae* homolog was capable of modifying murine H3 *and C. pneumoniae* HC1 (108). Whether these represent species-specific differences or are due to experimental differences remains to be determined. With the advent of genetic tools for manipulation of *C. trachomatis*, it will be interesting to determine whether NUE impacts host gene expression and plays a role in virulence.

*Legionella pneumophila* translocates over 300 proteins into the host cell during infection via its type IV secretion system (109). Of these, regulator of methylation A (RomA) contains a eukaryotic-like SET domain that allows it to mimic host methyltransferases, methylating histone 3 lysine 14 (H3K14) and causing a simultaneous decrease in histone acetylation, repressing gene expression (110) (Fig. 2C). RomA is also able to methylate 34 nonhistone proteins, suggesting a multifaceted role during infection (111). All sequenced strains of *L. pneumophila* encode a RomA homolog, with each homolog possessing at least one SET domain. Analysis of these orthologs revealed that the SET domain is always associated with an ankyrin domain (112). Crystallization of RomA in complex with S-adenosyl homocysteine and the N-terminal tail of histone H3 revealed that C-terminal ankyrin repeats form important contact sites, and these interactions are essential for histone methyltransferase activity (113). As histone H3 is usually acetylated, RomA must work in concert with an *L. pneumophila* histone deacetylase, LphD, to deacetylate H3K14 to allow for methylation by RomA (114). By using two distinct histone modifiers that work in synergy, the bacterium is able to strategically control the host response to infection.

## PATHOGEN MANIPULATION OF HOST TRANSCRIPTION VIA DIRECT DNA BINDING

Direct interaction with host DNA allows pathogens to control gene expression by mimicking transcription factors, binding to promoter regions, recruiting enzymes to modify histones, and altering chromatin structure. The study of viral transcription factors is an emerging field that will advance our understanding of host–pathogen interactions and the impact of viral infection on host gene expression (115). The multifunctional HIV protein Tat exemplifies this strategy by directly targeting host DNA through interactions with both promoter regions and introns (116) (Fig. 2D). Chromatin immunoprecipitation sequencing (ChIP-seq) analysis of ectopically expressed Tat and infected T-cells identified nearly 3,000 Tat-binding sites, correlating with the altered expression of approximately 2,000 host genes (116). Further analysis revealed that only a quarter of these are likely direct targets of Tat, which binds to promoter regions or mid-gene sites. In addition to its direct interaction with promoter regions, where it can both activate and repress gene expression, Tat also modulates RNA polymerase II activity. It can either initiate or block transcription at promoter regions or influence the elongation step by promoting or preventing the recruitment of positive transcription elongation factor b (pTEFb), which is essential for transcriptional elongation (116). Interestingly, several studies (116) (117) suggest a role for the host transcription factor ETS1 in Tat-mediated regulation. Tat preferentially binds to ETS1-regulated genes and co-immunoprecipitates with ETS1. Although more work is needed to delineate the mechanism(s) by which Tat regulates individual genes or gene clusters, these findings underscore the capacity of viral proteins to target host gene expression at multiple levels of regulation.

Multiple EBV proteins also directly bind host DNA, functioning as transcription factors (Fig. 2D). Recent work found that during a model of EBV reactivation, over 5,000 genes are downregulated, while more than 300 are upregulated (118). This downregulation of genes aligns with host transcription shut-off mechanisms that degrade mRNAs. However, the upregulated genes were of particular interest as sequenced transcripts had start sites outside of the known host start sites. These “*de novo”* transcripts were found to be driven by viral transcription factors, Zta and Rta, along with a viral preinitiation complex (vPIC), resembling that of gamma herpesviruses (118, 119). Future studies should investigate how this unique transcription mechanism shapes host–pathogen interactions.

*Anaplasma phagocytophilum*, *Ehrlichia chaffeensis*, and *Coxiella burnetii* secrete nucleomodulins that directly bind to host DNA. Of these *Rickettsial*-like pathogens, AnkA from *A. phagocytophilum* was the first secreted effector observed to localize to the nucleus and bind to DNA (120). Initial studies suggested that AnkA lacks a canonically defined NLS, but indicated that the N-terminus, which harbors Ank repeats, is necessary for AnkA import into the nucleus (121). New studies suggest AnkA possesses a classical NLS that overlaps Ank repeat 4. Once inside the nucleus, AnkA binds to AT-rich regions of DNA, where AnkA attaches to matrix attachment regions (MARs), inducing three-dimensional changes to the chromatin structure, effectively altering the transcriptional landscape (122). AnkA downregulates the expression of host genes, notably *CYBB*, through an interaction with transcriptional regulatory regions of the DNA (122) (Fig. 2D). AnkA binding to the *CYBB* promoter triggers recruitment of histone deacetylase 1 (HDAC1), resulting in deacetylation of histone H3 (121).

Similar to AnkA from *Anaplasma, E. chaffeensis* secretes the ankyrin repeat protein p200, which translocates to the nucleus of mononuclear host cells, where it binds to adenine-rich motifs within *Alu-Sx* elements, leading to altered expression of genes involved in apoptosis, transcription, cell signaling, and ATPase activity (123) (Fig. 2D). Although a specific DNA-binding domain has yet to be identified, p200 likely interacts either directly with chromatin or indirectly via host chromatin-associated proteins. *Alu-Sx* elements make up approximately ~10% of the human genome and are most commonly found near gene transcriptional start sites, enabling *E. chaffeensis* to induce widespread changes in host gene expression. In addition to p200, tandem repeat proteins (TPR) 32, TRP47, and TRP120 function as nucleomodulins (Fig. 2D). Although the precise nuclear entry mechanisms of these T1SS effectors remain largely unknown, TRP47 is predicted to utilize a “piggy-backing” strategy. Eukaryotic Linear Motif (124) analysis identified a myeloid, Nervy, DEAF-1 (MYND)-binding domain (MBD)—a motif that mediates interactions with transcriptional regulators—suggesting that TRP47 associates with an MYND domain-containing protein for nuclear localization (125). While the nuclear import method of TRP47 may differ from those of TRP32 and TRP120, all three proteins appear to bind host DNA via their tandem repeat domains. TRP32 requires phosphorylation of Y179 in its C-terminus to localize to the nucleus, where it binds G-rich motifs within the first 500 bp upstream of gene transcription start sites (81). TRP120, in contrast, binds to G + C rich regions (82). Together, TRP32, TRP47, and TRP120 illustrate how a pathogen can secrete related proteins with distinct functions and target sites, ultimately reprogramming host gene expression to establish an intracellular environment favorable to pathogenesis. Further research is essential to clarify the extent of TRP32, TRP47, and TRP120’s roles in epigenetically modulating host cells.

Of the *C. burnetii* effectors shown to localize to the nucleus, CBU1314 and NopA were shown to bind to chromatin and alter the host transcriptome (126–128). Recently, CBU1314 has been further characterized, showing it immunoprecipitates with host protein complex PAF1C, which is linked to transcriptional regulation including chromatin modification (128) (Fig. 2D). NopA is found in the chromatin fraction of cells during infection and alters nucleocytoplasmic transport through an interaction with Ran (127). Through this interaction, there is an accumulation of Ran-GTP, ultimately inhibiting nuclear import of transcription factors, leading to decreased expression of innate immune genes (127). These findings highlight the sophisticated mechanisms by which *C. burnetii* effectors manipulate host nuclear processes to suppress immune responses and promote bacterial survival.

Interestingly, the PAF1 complex is also targeted by viruses. IAV nonstructural protein 1 (NS1) contains a histone 3-like sequence, allowing it to function as a histone mimic (129). Affinity purification–mass spectrometry experiments revealed that NS1 tail peptides interact with the PAF1 complex, leading to the suppression of PAF1 complex-mediated transcriptional elongation (129). Furthermore, knockdown of *PAF1* results in decreased expression of antiviral genes (129). The importance of this interaction is further supported by experiments demonstrating that the removal of the NS1 binding site on PAF1 leads to attenuated IAV infection (129), highlighting how the virus targets PAF1 to alter host transcription to dampen the antiviral response (Fig. 2D). Similarly, dengue virus (DENV) protein NS5 was found to interact with components of the PAF1 complex, an interaction further shown to be conserved across flaviviruses (130). Initial work demonstrated that knockdown of the PAF1 complex led to increased DENV infectivity, while RNA-seq analysis revealed that NS5-expressing cells had reduced expression of ISGs (130) (Fig. 2D). Later work showed that PAF1 complex knockdown resulted in increased DENV virion production (131). These studies collectively highlight how viruses can hijack the PAF1 complex, preventing it from binding to the promoters of antiviral genes to facilitate infection.

## CONCLUSIONS

In this review, we have highlighted a subset of viral and bacterial proteins that manipulate host gene expression, often to suppress immune responses and facilitate pathogen replication. These examples illustrate the remarkable diversity of mechanisms pathogens use to regulate host gene activity, from disrupting nucleocytoplasmic transport at the nuclear pore complex to targeting transcriptional regulators like RNA polymerase II, altering lamin structure, directly binding DNA, and modifying histones post-translationally. By comparing the shared and distinct strategies of bacterial and viral nuclear targeting, we highlight the convergent evolutionary pressures driving these interactions. Future research is essential to further unravel the mechanisms underlying these host–pathogen interactions and their impact on transcriptional landscapes. Such insights will deepen our understanding of pathogen biology and identify potential therapeutic targets to counteract infection.
